# A Diagnostic Pitfall: Lupus Mastitis Presenting Before Diagnosis of Systemic Lupus Erythematosus

**DOI:** 10.1155/crrh/5593452

**Published:** 2026-02-17

**Authors:** Saeed Rashaad Mohammed, Kavi Capildeo, Keisha Davis-King, Mickhaiel Barrow

**Affiliations:** ^1^ Medical Associates Hospital, Saint Joseph, Trinidad and Tobago; ^2^ Department of Medicine, Faculty of Medical Sciences, University of the West Indies, St. Agustine Campus, Saint Augustine, Trinidad and Tobago, uwi.edu; ^3^ Department of Medicine, Eric Williams Medical Sciences Complex, North Central Regional Health Authority, Champs Fleurs, Trinidad and Tobago, ncrha.co.tt; ^4^ Department of Pathology, Port of Spain General Hospital, North West Regional Health Authority, Port of Spain, Trinidad and Tobago, health.gov.tt

## Abstract

Lupus erythematosus panniculitis or lupus erythematosus profundus (LEP) is a rare manifestation of cutaneous lupus erythematosus, with an estimated prevalence of 2%–3% in those with either systemic lupus erythematosus (SLE) or discoid lupus erythematosus (DLE). LEP with involvement of the breasts is termed lupus mastitis (LM). Its presentation is heterogenous, with epidermal changes, erythema, violaceous skin changes, lipoatrophy, and ulceration with or without breast masses. LM may resemble breast malignancy; however, the clinical course, laboratory investigations, and imaging may often differentiate these. Should uncertainty still exist, LM may be confirmed on histopathology. LM is a chronic disease, and its natural course may include exacerbations and remissions. Antimalarial agents are the mainstay of treatment, whilst corticosteroids and cyclophosphamide have demonstrated utility. There is no standardized treatment protocol. We here present the case of a 50‐year‐old woman who was diagnosed with SLE and LM after several indeterminate breast biopsies with the intention of furthering awareness of this presentation.

## 1. Introduction

Inflammation of the subcutaneous adipose tissues is a possible manifestation of several connective tissue disorders [1]. This panniculitis is typically classified based on the underlying disease. Lupus erythematosus panniculitis is a rare manifestation of cutaneous lupus erythematosus, first described in 1883 by Kaposi [2], whilst the term lupus erythematosus profundus (LEP) was introduced by Irgang in 1940 [3]. There is inconsistent application of these terminologies across the literature, with some using them synonymously, whilst others utilize lupus panniculitis to describe subcutaneous inflammation in the absence of overlying cutaneous manifestations [4, 5].

LEP can occur in patients with either systemic lupus erythematosus (SLE) or discoid lupus erythematosus (DLE) and has an estimated prevalence of 2%–3% [6–8]. Those with LEP develop tender subcutaneous nodules or plaques across the face, shoulders, hips, buttocks, and proximal extremities [1, 9]. Lupus mastitis (LM) refers to LEP with involvement of the mammary gland/breasts [10]. Clinical presentation of LM is heterogenous; epidermal changes may be observed (erythema, violaceous skin changes, lipoatrophy, or ulceration), with or without the observation of palpable breast masses [11]. Prominent skin changes may provoke suspicion of breast malignancy, leading to unnecessary investigations.

The authors here present a case of LM, diagnosed after numerous indeterminate breast biopsies.

## 2. Case Presentation

A 50‐year‐old presented to an institution with a four‐month history of vertigo, dyspnea, orthopnea and peripheral edema to the mid‐thighs. She reported a 3‐month history of bilateral mastalgia with associated swelling and erythema of the breasts. The past medical history was significant only for hypertension for the past 16 years, managed with oral antihypertensives.

She had presented to another institution 1 month after onset of initial symptoms; complete blood count at that time revealed pancytopenia prompting hematology review; further investigations noted elevated immunoglobulins (Immunoglobulin G 4025 mg/dL [reference range: 700–1600], Immunoglobulin M 88 mg/dL [reference range: 40–230], and Immunoglobulin A 583 mg/dL [reference range: 70–400]). This led to a bone marrow aspiration and biopsy. Accompanying complete blood count revealed a slight normochromic normocytic anemia (hemoglobin: 10 g/dL), leukopenia with neutropenia (1200/μL), and moderate thrombocytopenia (79,580/μL). Marrow was hypercellular with trilineage hematopoiesis including erythroid and megakaryocytic hyperplasia with morphologic atypia and slight reticulin fibrosis. There was marked polyclonal plasmacytosis, deemed reactive.

Several weeks following bone marrow biopsy, the patient noted bilateral mastalgia and swelling of the breasts. Breast ultrasound demonstrated skin thickening and diffuse edema with dilated ducts in bilateral retroareolar regions. A provisional diagnosis of nonspecific mastitis was made, and the patient was referred to the surgical outpatient clinic. A core‐needle biopsy of the right breast was performed, displaying nonspecific features with fibrous and adipose tissues with focal hemorrhage and mild, patchy chronic inflammation. The patient noted marked skin changes on both breasts over the next several weeks, leading to the surgical team performing core‐needle biopsies of both breasts, performed 6 weeks after the first biopsy.

**FIGURE 1:**
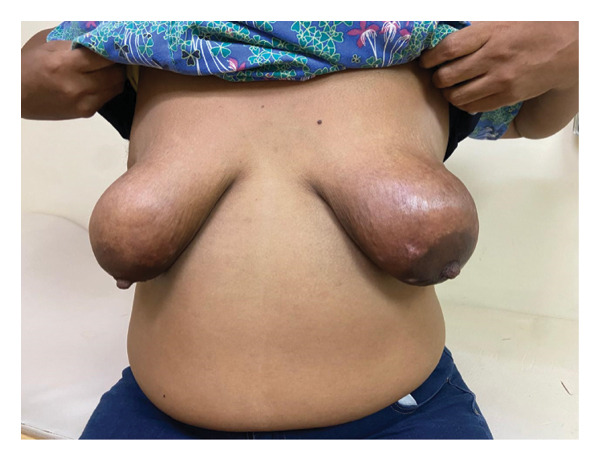
Hyperpigmentation of the bilateral breasts.

**FIGURE 2:**
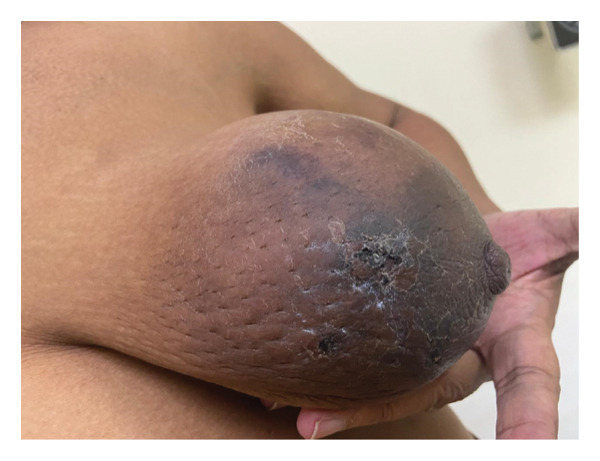
Ulceration of the left breast.

The patient presented to the hematology/oncology clinic at our institution while awaiting the results of these biopsies. Examination revealed bilateral hyperpigmentation of the breasts (Figure 1) with ulceration of the skin (Figure 2).

Serum protein electrophoresis (SPEP) and computed tomography (CT) scan of the head, chest, abdomen, and pelvis were performed.

CT imaging revealed mosaic attenuation within the bilateral lung parenchyma, chronic portal vein thrombosis extending from the superior mesenteric vein through the portal vein branches, and splenomegaly. There were multiple enlarged retroperitoneal, pelvic, and inguinofemoral lymph nodes and generalized anasarca and edema of the breasts. SPEP displayed polyclonal hypergammaglobulinemia, with possible Beta‐2 gamma bridging.

Pathological evaluation of the breast biopsies became available at this stage; the right breast biopsy again revealed mature fibroadipose tissue with inflammatory cell infiltrates and evidence of fat necrosis. The left breast biopsy displayed fibroadipose tissue and evidence of previous infarction. Cytological details were poorly preserved, and a repeat left breast biopsy was recommended.

A clinical diagnosis of panniculitis was made, and an autoimmune panel was requested. Antinuclear antigen (ANA) (3.140, positive > 1.2), anti–double‐stranded deoxyribonucleic acid (Anti‐dsDNA) antibodies (92.697 U/mL, positive ≥ 25), and anti‐SSA/Ro (1.731, positive > 1.2) were positive, whilst anti‐SSB/La (0.442, positive > 1.2) and anti‐Smith (1.117, positive > 1.2) were negative.

The diagnosis was established as SLE with LM, and treatment was initiated with hydroxychloroquine 200 mg orally once daily, mycophenolate mofetil 500 mg orally twice daily, and prednisolone 50 mg orally once daily for a 2‐week course. Warfarin treatment was begun and titrated to the appropriate dosing regimen.

Subsequently, warfarin was discontinued in favor of subcutaneous enoxaparin sodium, prednisolone was tapered and discontinued, and mycophenolate mofetil dose increased to 1g orally twice daily.

## 3. Discussion

LEP is most frequently observed in persons with a known diagnosis of SLE or DLE [12], although there are instances of it being the presenting symptom [10, 12]. There are approximately 50 cases of LM reported in the English literature, with the primary affected demographic being women aged 40–50 years [9, 12]. The case presented herein highlights LM presenting prior to the diagnosis of SLE, resulting in multiple nondiagnostic biopsies and diagnostic delay. The patient’s initial presentation was systemic and hematologic abnormalities; thus, an underlying autoimmune etiology was not considered at first. Subsequently, histopathologic findings were reported as nonspecific fat necrosis, further delaying recognition of the underlying disease.

The exact pathophysiology of LEP is yet to be fully elucidated, with current understanding attributing it to an autoimmune inflammatory process that involves the subcutaneous tissue [6, 9, 13]. Linear deposition of immunoglobulin M and C3 along the dermoepidermal junction and vessel basement membrane and symptomatic improvement upon commencement of immunosuppressive therapy form the basis of this theory [6]. Early reports suggested that LEP may be incited or exacerbated by trauma [14], but this has since been disputed [5, 9]. Indeed, the patient reported herein did not note any worsening skin symptoms subsequent to her biopsies.

LM may present as epidermal changes or breast masses, either alone or concomitantly. Differential diagnoses include benign masses, inflammatory breast conditions, and breast malignancy. Should LM present prior to a diagnosis of SLE or DLE being established, it is logical to proceed to radiologic and histopathological investigations.

Mammography, ultrasound, and magnetic resonance imaging (MRI) may be employed, either singly or in combination [9, 10]. Their utility is hampered by the limited number of precise descriptions of their findings [10]. Mammographic descriptions note ill‐defined asymmetric masses corresponding to palpable lumps, extensive skin thickening, and dense breast tissue [6, 7]. The extent of calcifications is widely variable, from fine linear branching microcalcifications [15] to large, coarse calcifications indicative of fat necrosis [10, 13]. Ultrasound demonstrates similar findings, alongside ill‐defined masses of variable echogenicity, with or without increased blood flow on color Doppler [12], and possible axillary lymphadenopathy [13]. MRI displays nonspecific findings, which may yet assist in the diagnosis. The presence of irregular masses with rim enhancement may trigger suspicion of fat necrosis, whilst T1‐weighted imaging can identify internal fat components [15]. A hyperintense signal and pronounced fat stranding on the T2‐weighted sequence are other notable features [10, 12].

LM may be confirmed on histopathology. Four major and four minor criteria have been put forth as consistent with a diagnosis of LM [4, 5]. Major criteria are hyaline fat necrosis, lymphocytic infiltration with lymphoid nodules surrounding the necrosis, periseptal or lobular panniculitis, and microcalcifications [4, 5]. Minor criteria are changes of DLE in the overlying skin, lymphocytic vascular infiltration, mucin deposition, and hyalinization of subepidermal papillary zones [4, 5]. Not all criteria need to be observed to facilitate the diagnosis. Immunohistochemistry, although rarely performed, shows a mixed population of CD20^+^ B‐lymphocytes and predominantly CD3^+^ and CD4^+^ T‐lymphocytes along with polyclonal plasma cells [7].

The clinical course, laboratory investigations, imaging, and histopathology are usually sufficient to make an accurate diagnosis [6, 10]. It is most crucial that a diagnosis of breast malignancy be excluded. Whilst inflammatory breast cancer (IBC) may resemble LM on imaging, it is typically painful, with the skin indurated and resembling peau d’orange [16]. IBC is usually unilateral and rapidly progressive, unlike the majority of cases of LM [6, 16]. Histology of IBC is characterized by dermal lymphatic infiltration of tumor cells without inflammation, thus differentiating it from LM [6, 16].

Lymphoma can, on rare occasions, involve the breast [6, 9]. Whilst diffuse large B‐cell lymphoma is most common [17], subcutaneous panniculitis‐like T‐cell lymphoma (SPTL) of the breast has been infrequently reported [18]. The histology may possess overlapping features with LM, and if the diagnosis is unclear, immunohistochemistry and molecular studies are warranted [9]. The examination of intramammary and regional lymph nodes may provide additional information; bilaterally enlarged lymph nodes increase suspicion of non‐Hodgkin’s lymphoma [6, 17].

Several benign breast diseases may resemble LM, including diabetic mastopathy and idiopathic granulomatous mastitis. Diabetic mastopathy should be considered in patients with Type 1 diabetes [7, 19]. Dense periductal, perilobular and perivascular lymphocytic infiltrates are prominently featured in diabetic mastopathy, whilst fat necrosis is not considered a characteristic feature, differentiating it from LM [7, 19].

Idiopathic granulomatous mastitis has a similar clinical presentation to LM: skin erythema, firm breast masses, swelling, and mastalgia [20]. Breast ultrasound demonstrates hypoechoic masses with indistinct or irregular margins and reactive appearing regional nodes [21]. Histology is characterized by non‐necrotizing granuloma formation in breast lobules, while necrosis is rare [21]. The absence of granulomatous inflammation on multiple biopsies excluded this diagnosis in the patient reported herein.

LM is usually diagnosed in the setting of known SLE or DLE. The diagnosis may be delayed in cases such as that presented here, where a diagnosis of SLE/DLE has not been made previously. Whilst the histopathologic features may render the diagnosis when a background diagnosis of SLE or DLE is known, they may be more generically defined as “lobular panniculitis” in the absence of such [7].

LM is a chronic disease with exacerbations and remissions. Chronic untreated LM may result in severe lipoatrophy, scarring, and ulceration [6, 8]. Antimalarial agents are the primary treatment option, whilst severe LM has been successfully treated with corticosteroids or cyclophosphamide [6, 8]. The rarity of the disease precludes a standardized treatment regimen.

This case emphasizes that LM should be considered in patients with inflammatory breast changes and repeated nondiagnostic biopsies, even in the absence of a prior diagnosis of SLE or DLE. Increased awareness may prevent unnecessary biopsies and expedite initiation of appropriate treatment.

## 4. Conclusion

LM is a rare presentation of SLE/DLE and may pose a diagnostic dilemma if it is the first presentation of the underlying disease. A patient may oftentimes be undergoing workup for multiple complaints/symptoms, hence a multidisciplinary approach may assist in identifying the underlying systemic disease. LM may often incite concern for a breast malignancy; thus, thorough history‐taking and breast imaging may aid in diagnosis. Should these not be sufficient, breast biopsies may be warranted; early reports of biopsies precipitating further skin changes have been disputed in recent years.

## Author Contributions

Saeed Rashaad Mohammed and Kavi Capildeo conceptualized the manuscript. All authors contributed to drafting the manuscript and to subsequent revisions. Kavi Capildeo provided supervision throughout.

## Funding

No funding was received for this study.

## Disclosure

All authors have read and approved the final manuscript.

## Ethics Statement

Due to the present study being a retrospective case report, ethical approval was not required. Informed consent was obtained from the patient for all procedures conducted as part of clinical management.

## Consent

Written consent was obtained from the patient and is available upon editorial request.

## Conflicts of Interest

The authors declare no conflicts of interest.

## Data Availability

The data that support the findings of this study are available from the corresponding author upon reasonable request.
